# Lactogenic differentiation of HC11 cells is not accompanied by downregulation of AP-2 transcription factor genes

**DOI:** 10.1186/1756-0500-1-29

**Published:** 2008-06-23

**Authors:** Richard Jäger, Leontios Pappas, Hubert Schorle

**Affiliations:** 1 Institute for Pathology, Department of Developmental Pathology, University of Bonn Medical School, Sigmund-Freud-Strasse 25, 53127 Bonn, Germany; 2 Harvard University, Faculty of Arts and Sciences, Cambridge 02138, MA, USA

## Abstract

**Background:**

During pregnancy the mammary epithelium undergoes a complex developmental process which culminates in the generation of the milk-secreting epithelium. Secretory epithelial cells display lactogenic differentiation which is characterized by the expression of milk protein genes, such as beta-casein or whey acidic protein (WAP). Transcription factors AP-2alpha and AP-2gamma are downregulated during lactation, and their overexpression in transgenic mice impaired the secretory differentiation of the mammary epithelium, resulting in lactation failure. To explore whether the downregulation of AP-2alpha and AP-2gamma is of functional significance for lactogenic differentiation, we analyzed the expression of the AP-2 family members during the lactogenic differentiation of HC11 mammary epithelial cells in vitro. Differentiation of HC11 cells was induced following established protocols by applying the lactogenic hormones prolactin, dexamethasone and insulin.

**Findings:**

HC11 cells express all AP-2 family members except AP-2delta. Using RT-PCR we could not detect a downregulation of any of these genes during the lactogenic differentiation of HC11 cells in vitro. This finding was confirmed for AP-2alpha and AP-2gamma using Northern analysis. Differentiating HC11 cells displayed lower expression levels of milk protein genes than mammary glands of mid-pregnant or lactating mice.

**Conclusion:**

The extent of lactogenic differentiation of HC11 cells in vitro is limited compared to mammary epithelium undergoing secretory differentiation in vivo. Downregulation of AP-2 transcription factor genes is not required for lactogenic differentiation of HC11 cells but may functionally be involved in aspects of lactogenic differentiation in vivo that are not reflected by the HC11 system.

## Background

During pregnancy the mammary epithelium matures in a complex developmental process which culminates in the generation of alveoli containing the milk-secreting epithelium. The lactogenic differentiation of the secretory epithelial cells is characterized by the synthesis of lactose and milk fat and by the expression of milk protein genes, such as beta-casein or whey acidic protein (WAP). The regulatory networks and transcription factors controlling these developmental processes are only incompletely understood [1,2].

Transcription factors of the AP-2 family comprise 5 members, termed AP-2alpha-epsilon (or TFAP2a-e), which control genetic programs involved in proliferation, apoptosis, and differentiation (see [3] for review). They share a conserved structure and control gene expression by binding to the respective promoters as homo- or heterodimers. Transcription factors AP-2alpha and -gamma have been implicated in breast cancer [4] and, based on overexpression in transgenic mice, in mammary development [5,6]. AP-2alpha and -gamma are expressed in non-pregnant mammary epithelium and are downregulated in lactating mammary glands. Overexpression of AP-2alpha or -gamma has been shown to interfere with the proper secretory differentiation of mammary epithelial cells at the end of pregnancy, resulting in lactation failure [5,6]. These findings suggest a functional role of AP-2 transcription factors in preventing lactogenesis. To date, the genetic programs mediating this effect have not been identified. To these ends, an in vitro system of lactogenic differentiation would be informative.

The murine mammary epithelial cell line HC11 represents an established in vitro system for the study of lactogenic differentiation [7,8]. HC11 cells can be induced to undergo lactogenic differentiation by applying a mixture of the lactogenic hormones dexamethasone, insulin and prolactin (a mixture termed DIP in the following sections). In this report we analyzed AP-2 transcription factor expression in HC11 cells subjected to lactogenic differentiation conditions.

## Methods

HC11 cells were grown and differentiated as described in [9]. Hormones were obtained from Sigma (Hamburg, Germany). Routine culture was performed using RPMI 1640 medium containing 10% fetal calf serum, L-glutamine, 5 μg/ml Insulin and 10 ng/ml epidermal growth factor (EGF). For induction of differentiation, cells were grown to confluency and then kept in medium without EGF for 48 hours to induce competence. Competent cells were incubated with DIP medium (RPMI 1640 medium containing 10% FCS, L-glutamine, 100 nM dexamethasone, 5 μg/ml insulin, 5 μg/ml ovine prolactin) for 72 hours.

RNA was prepared from confluent cultures or mammary tissue as described previously [5]. 1 μg of total RNA was reverse-transcribed using AMV reverse transcriptase (Invitrogen, Karlsruhe, Germany) and Oligo (dT)_20 _primers following the manufacturer's instructions. Primers used for subsequent PCR are listed in Table 1. The primers used to detect AP-2 family members amplify regions within the non-conserved 3'-untranslated regions of the respective mRNA.

**Table 1:** Oligonucleotides used for PCR

cDNA	Primer pair	no. of cycles
β-actin	5'-CCATCCTGCGTCTGGACCTG-3' 5'-GTAACAGTCCGCCTAGAAGC-3'	25
β-casein	5'-CCATCCTGCGTCTGGACCTG-3' 5'-GGAATGTTGTGGAGTGGCAG-3'	25
WAP	5'-AAAAGCCAGCCCCATTGAGG-3' 5'-AGGGTTATCACTGGCACTGG-3'	30
AP-2α	5'-CCACTCCTACTGCTGCTGCTACTCT-3' 5'-GTTCACAAACGCGACAGAACTTTTC-3'	30
AP-2β	5'-TAACATATTGGATTGGCTTTGGAGG-3' 5'-CTCACGTTTTATTTTCCAAAAGGGG-3'	35
AP-2γ	5'-ATCACACCTTCTGGTAGGAGGCAG-3' 5'-AGGCTTAGAGGTCCAGTCCCAATC-3'	30
AP-2δ	5'-AATCTATTTCCAGAGAGTCTTGCTGC-3' 5'-GATTTCATTTTATGGAGGGCTCAGG-3'	30
AP-2ε	5'-AAATGTGGGATTCTATTCAGGCCAG-3' 5'-ACAGGACTGTGGTAATGCAGCCTA-3'	30

Northern Blot analyses were carried out as previously described [5]. Briefly, 15 μg of total RNA was resolved on formaldehyde agarose gels, transferred to positively charged nylon membrane (Hybond N^+^, Amersham, Braunschweig, Germany), hybridized to ^32^P-labelled DNA probes and washed under stringent conditions (0.1 × SSC/65°C). The AP-2 probes used cover nucleotides 1760–2040 from the murine AP-2alpha and 2041–2710 from the murine AP-2gamma mRNA, respectively, both sequences within the non-conserved 3'-untranslated regions. WAP, beta-casein, and GAPDH probes have been described previously [5].

## Results and Discussion

**Figure 1:**
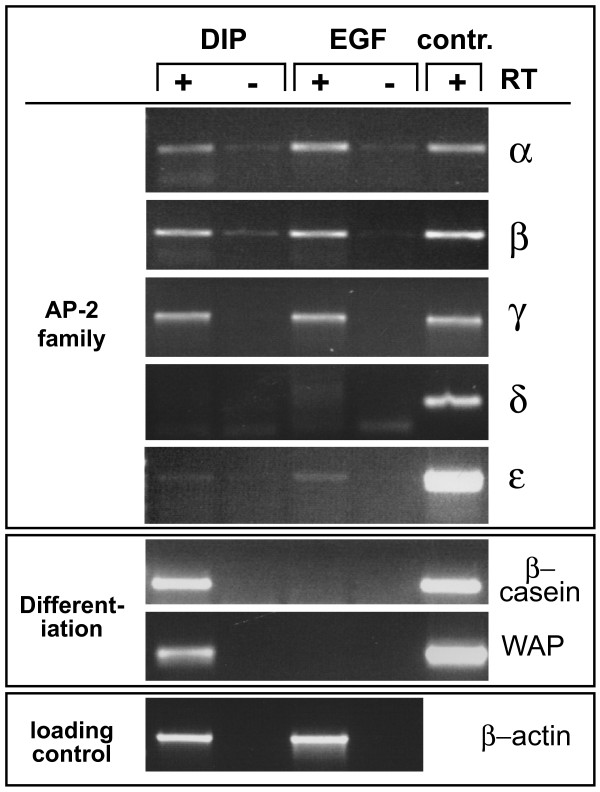
**Expression of AP-2 family members in HC11 cells**. RNA from HC11 cells cultivated in EGF-containing medium (EGF) or in differentiation conditions (DIP) was subjected to RT-PCR using primers specific for the AP-2 isoforms indicated (upper panel) or milk protein genes (middle panel). Amplification of beta-actin served as a loading control (lower panel). Control reactions were carried out in the absence of reverse transcriptase (-RT). Positive controls (contr.) consisted of cloned PCR fragments (10 pg plasmid DNA) in the case of AP-2 isoforms, or of cDNA derived from a lactating mouse mammary gland for differentiation markers.

To assess AP-2 transcription factor gene expression, HC11 cells were grown under normal conditions (in the presence of EGF) or subjected to differentiation conditions by first culturing to confluency without EGF and then treatment with the lactogenic hormones dexamethasone, insulin and prolactin (DIP) for 72 hours. RNA was isolated from confluent cultures and subjected to RT-PCR analysis. As shown in Figure 1, only cells cultivated with DIP expressed the milk protein genes beta-casein and WAP, indicative of lactogenic differentiation (Figure 1, middle panel). HC11 cells expressed predominantly AP-2alpha, -beta, and -gamma, and at very low levels AP-2epsilon, whereas AP-2delta transcripts were not detectable. By RT-PCR we did not detect changes in expression of these AP-2 isoforms between non-differentiated and differentiated cells (Figure 1, upper panel). Therefore, AP-2 transcript levels do not appear to decrease during lactogenic differentiation of HC11 cells.

**Figure 2:**
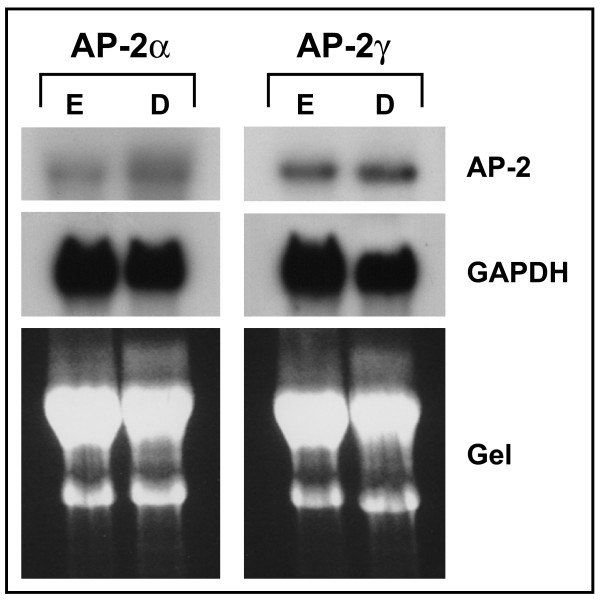
**Northern analysis of AP-2alpha and AP-2gamma expression**. RNA isolated from HC11 cells cultured in presence of EGF (E) or of cells differentiated with DIP (D) was gelelectrophoretically separated, transferred to a nylon membrane, and hybridized with the probes indicated. GAPDH: Glyceraldehyde-3-phosphate dehydrogenase. A gel photo is shown to demonstrate equal loading and integrity of RNA.

To verify this result, we tested the expression levels of AP-2alpha and AP-2gamma in a semi-quantitative manner using Northern blot analysis. We were particularly interested in these two AP-2 isoforms because both are downregulated during lactation in vivo, and overexpression of either isoform in the mammary glands of transgenic mice was interfering with lactation, suggesting a functional role in lactogenic differentiation [5,6]. As depicted in Figure 2, neither AP-2alpha nor AP-2gamma transcript levels decreased during lactogenic differentiation of HC11 cells. Taking into acount a decrease in GAPDH expression (relative to the amount of RNA shown by the gel photo), rather a slightly increased expression level of both AP-2 transcription factors during differentiation was observed. These results suggest that the lactogenic differentiation of HC11 cells does not require a downregulation of AP-2 transcription factors.

**Figure 3:**
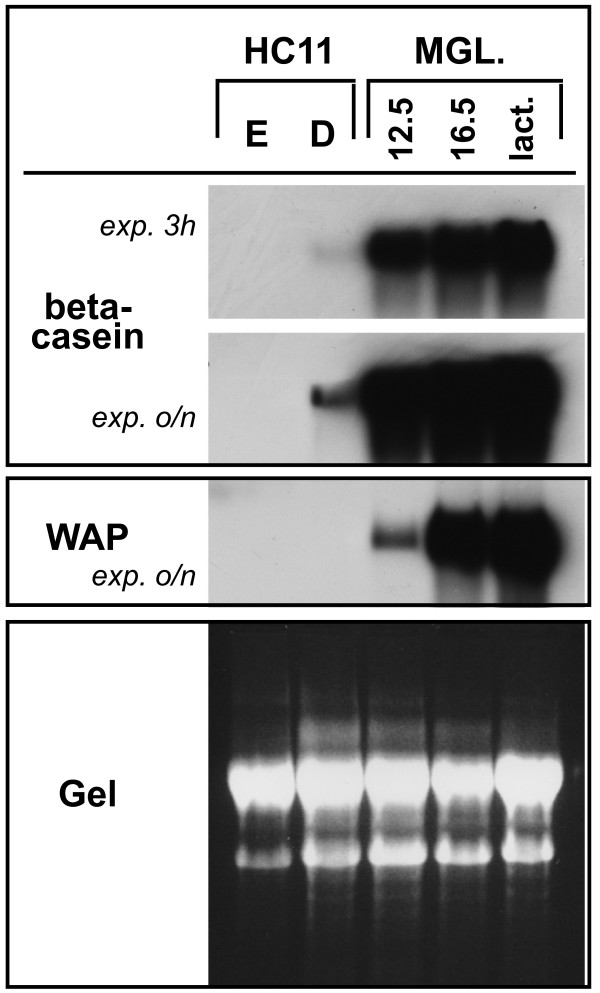
**Milk protein gene expression of HC11 cells and of mammary glands**. RNA of HC11 cells grown with EGF (E) or of differentiated cells (D) and of mouse mammary glands (MGL.) taken at day 12.5 or day 16.5 of pregnancy or at lactation was analyzed by Northern Blot. Upper panel: beta-casein gene expression. Films were exposed either for 3 hours (exp. 3 h) or overnight (exp. o/n). Middle panel: WAP gene expression, exposure was overnight (o/n). Lower panel: photo of the RNA gel to demonstrate amounts and integrity of the blotted RNA.

Because these findings were suggesting that differentiated HC11 cells are not representative of mammary epithelium at lactation, we compared milk protein gene expression levels between differentiated HC11 cells and mammary glands taken from mid- or late-pregnant mice or from lactating mice. Using Northern blot analysis, we could readily detect beta-casein transcripts in DIP-treated HC11 cultures. Their amount, however, was much lower than even in mammary glands at day 12.5 of pregnancy (Figure 3, upper panel). Whereas WAP transcripts could be readily amplified by RT-PCR (Figure 1) they were not detectable by Northern analysis in DIP-treated HC11 cells (Figure 3, middle panel). The kinetics of WAP gene expression in vivo is known to be delayed compared to beta-casein expression [10], and accordingly we could detect only low expression levels at day 12.5 of pregnancy. In conjunction with the sustained AP-2 transcript levels, these results clearly demonstrate that lactogenic differentiation of HC11 cells does not fully reflect the in vivo situation. Lactogenic differentiation in vivo can be subdivided into distinct phases [2]: First, secretory differentiation of alveolar epithelial cells is taking place which is characterized by increased biosynthesis of milk proteins and of lipids that are organized in membrane bound milk fat globules residing in the cytoplasm. The transition to actual milk secretion has been termed "secretory activation" and is reflected by a strong induction of genes involved in lipid biosynthesis. Whereas differentiating HC11 cells display milk protein gene induction and also secretory activation [1], several glycoproteins of the milk fat globule membrane are not appropriately expressed [11]. Moreover, expression of beta-Casein and of Xanthine oxidoreductase, a component of the milk fat globule, were shown to be controlled by distinct mechanisms in HC11 cells [12]. It therefore remains possible that the downregulation of AP-2 transcription factor genes plays a role in milk fat globule formation or other aspects of lactogenic differentiation that are not reflected by the HC11 system.

## Competing interests

The authors declare that they have no competing interests.

## Authors' contributions

RJ designed experiments, performed the Northern analyses and wrote the manuscript. LP performed cell culture experiments and RT-PCR analyses. HS intellectually contributed to the design and evaluation of the experiments and helped draft the manuscript.
